# Pharmacological rescue of social deficits in rats featuring Disrupted-in-Schizophrenia-1 (DISC1) protein aggregation

**DOI:** 10.1038/s41537-026-00729-y

**Published:** 2026-02-04

**Authors:** José Dören, Else Van Gerresheim, Sandra Schäble, Svenja Troßbach, Ann-Christin Langen, Heike Schneider, Werner Steimer, Tobias Kalenscher, Carsten Korth

**Affiliations:** 1 Comparative Psychology, Institute of Experimental Psychology, Heinrich Heine University Düsseldorf, Universitätsstraße 1, 40225 Düsseldorf, Germany; 2 Department of Neuropathology, Medical Faculty, Heinrich Heine University Düsseldorf, Universitätsstraße 1, 40225 Düsseldorf, Germany; 3 Department of Rheumatology & Hiller Research Center Rheumatology, Medical Faculty, Heinrich Heine University Düsseldorf, Universitätsstraße 1, 40225 Düsseldorf, Germany; 4 Institute for Clinical Chemistry and Pathobiochemistry, University Hospital Technical University, Ismaninger Strasse 22, 81675 Munich, Germany

**Keywords:** Schizophrenia, Diseases of the nervous system

## Abstract

The pharmacological treatment of negative symptoms in schizophrenia remains a major unmet need. Among these, impairments in social functioning – manifesting as reduced adaptability and social withdrawal – are particularly disabling, as they persist beyond remission of positive symptoms and impede social reintegration. To investigate the neurobiological basis of behavioral impairments, we employed the tgDISC1 rat, a translational model overexpressing the human non-mutant Disrupted-in-Schizophrenia-1 (DISC1) gene. This overexpression results in DISC1 protein aggregation and aberrant signaling– molecular features identified in a subset of schizophrenia patients identified by elevated DISC1 aggregates in cerebrospinal fluid. Behaviorally, the tgDISC1 rats exhibited a selective loss of social novelty preference in the 3-Chamber task while maintaining intact social interest, indicating a specific deficit in social adaptability rather than social motivation. Here, we tested whether continuous administration of atypical antipsychotics amisulpride or clozapine would rescue social deficits in tgDISC1 rats. Treatment with amisulpride (0.2 and 0.8 mg/kg/day for two weeks) fully restored social novelty preference, whereas clozapine had no effect. Control tasks for anhedonia, short-term working memory, and explorative behavior confirmed that their phenotype was not secondary to global motivational or cognitive impairments. Together, these findings demonstrate that amisulpride, a selective D2/D3 receptor antagonist, rescues social adaptability deficits linked to aberrant DISC1 signaling. The results also highlight the success of our precision psychiatry approach: the biological definition of a subset of schizophrenia by identifying DISC1 protein aggregates, the generation of a corresponding animal model and a successful pharmacotherapy of a clinically relevant phenotype.

## Introduction

Translational animal models are essential for capturing mechanistic insights of clinically relevant behavioral disorders in psychiatry, yet their utility critically depends on the degrees to which they resemble feature of patients. Model validity is therefore commonly distinguished into construct validity, reflecting correspondence with disease relevant biological mechanisms and markers; face validity, denoting similarities in behavioral impairments; and predictive validity, defined by clinically meaningful treatment responsiveness^1^.

In schizophrenia research, achieving convergent validity across these dimensions remains particularly challenging, given the disorder’s heterogeneity of underlying biological causes and that many core symptoms – especially negative and social impairments – lack sufficient mechanistic understanding^2,3^. Consequently, preclinical models often reproduce only isolated phenotypes, where molecular pathology, behavior or drug responsiveness are not integrated into a coherent translational framework^4^. Given this situation, animal models that allow systematic validation across these complementary levels are therefore needed for advancing the understanding of underlying pathological mechanisms and the transformation to therapeutic output.

Among these dimensions, predictive validity is of particular relevance for models aiming to inform treatment strategies for schizophrenia. To this end, classical antipsychotic drugs targeting mainly dopamine 2 receptors (D2R), but cross-reacting with many other neurotransmitter systems to various proportions, remain the cornerstone of clinical management^5^. Likewise, their efficacy differs markedly between symptom domains, with negative symptoms and social impairment showing limited and highly variable responsiveness^6^. Establishing predictive validity in preclinical models therefore captures a valuable opportunity to investigate differentiated, symptom-relevant responses and to extend stratified, biomarker-based therapeutic approaches.

**Fig. 1:**
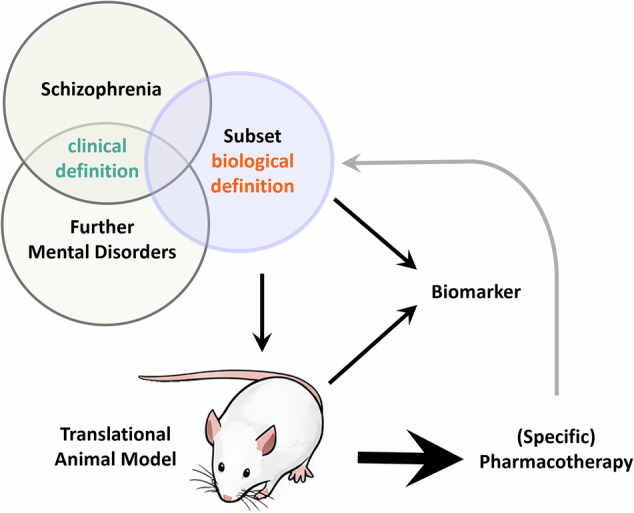
Reverse-translational precision psychiatry framework. Schematic representation of a reverse-translational precision psychiatry framework, in which identification of a biologically defined subset of schizophrenia informs the development of a face-valid animal model, enables the discovery of additional biomarkers, and guides the identification of effective pharmacotherapies that may ultimately be translated back to the original schizophrenia subset.

In this context, the present animal model is grounded in a molecular pathology observed in a defined subset of schizophrenia patients. Over the last decade we have established a unique reverse-translational, precision psychiatry approach consisting in biologically defining a subset of patients with schizophrenia, identifying ante mortem biomarkers, a corresponding face valid animal model, and, ultimately, investigating tailored pharmacotherapies, validated, ideally, in both the animal model and the patient subset (Fig. 1).

We started by defining a subset of patients with chronic mental diseases including schizophrenia by identifying protein aggregates of a critical behavior-relevant signaling molecule Disrupted-in-Schizophrenia-1 (DISC1) protein in post-mortem brain tissue^7,8^, and later in the cerebrospinal fluid of first-episode psychotic patients^9^. Protein biochemical studies established a high aggregation propensity of DISC1 protein^7,10^ narrowing functional expression levels between too low (as in the familial cases due to haplinsufficiency^11^) and too much (leading to aggregation^7^).

To model this molecular pathology, transgenic DISC1 (tgDISC1) rats that modestly overexpress the human non-mutant DISC1 gene featured aberrant protein aggregation and downstream signaling disturbances^12^. At the molecular level, tgDISC1 rats exhibit an increased proportion of high-affinity D2R alongside dopamine transporter translocations and aberrant dopamine neuroanatomy, establishing dopaminergic dysregulation as a central pathophysiological mechanism^12,13^. Importantly, these molecular alterations translate to broader structural changes: recent diffusion tensor imaging revealed microstrucutal abnormalities in mesolimbic and associative brain regions which are critically involved in dopaminergic signaling and frequently implicated in schizophrenia^14–18^.

At the behavioral level, tgDISC1 rats demonstrate behavioral alterations – most consistently – impairments in social behavior across different experimental paradigms^12,18–20^. Notably, these behavioral abnormalities are not shaped by global reduction of motivation but rather selective impairments in social adaptability, such as deficits in social reward learning or a lack of social novelty preference (in both sexes)^18,20^. The specificity of these social symptoms and the absence of generalized cognitive impairment underscores their relevance for studying discrete aspects of negative symptoms in schizophrenia. Together, this constellation of a defined molecular pathology alongside selective behavioral deficits provides a valuable opportunity for probing pharmacological responsiveness to antipsychotic treatment.

Here, we examined whether social behavioral alterations in male tgDISC1 rats are pharmacological reversible by continuous administration of second-generation antipsychotics amisulpride or clozapine. Both drugs are frequently used in clinical therapeutics but differ in their receptor-profile. Clozapine covers affinity to D2R, D3R and D4R, as well as serotonin, noradrenalin and adrenergic receptors (among others) and remains the gold-standard for treatment resistant schizophrenia^21^. In contrast, amisulpride is a rather selective (but not exclusive) D2R/D3R antagonist^22^. Using subcutaneous osmotic pumps to achieve stable drug exposure, animals underwent a behavioral test battery with a particular focus on social interaction and social novelty preference in the 3-Chamber task, while controlling for locomotor activity, hedonic drive and cognitive performance. This study aimed to further delineate the translational relevance of the tgDISC1 model and to evaluate its predictive validity with respect to clinically relevant therapeutics.

## Results

### Behavior

In order to investigate the effects of antipsychotics amisulpride and clozapine on social behavior we conducted the 3-Chamber task. We also conducted multiple control tasks to ascertain that the drug effects on behavior in the 3-Chamber task could not be explained by differences between genotypes and drug conditions in hedonic drive, working memory, or behavioral inflexibility, such as rigid exploration patterns. To control for a general deficit in reward-related behaviors, we compared sucrose consumption in all animals but did not find differences (Supplemental Fig. S1A).

#### Social novelty preference

**Fig. 2:**
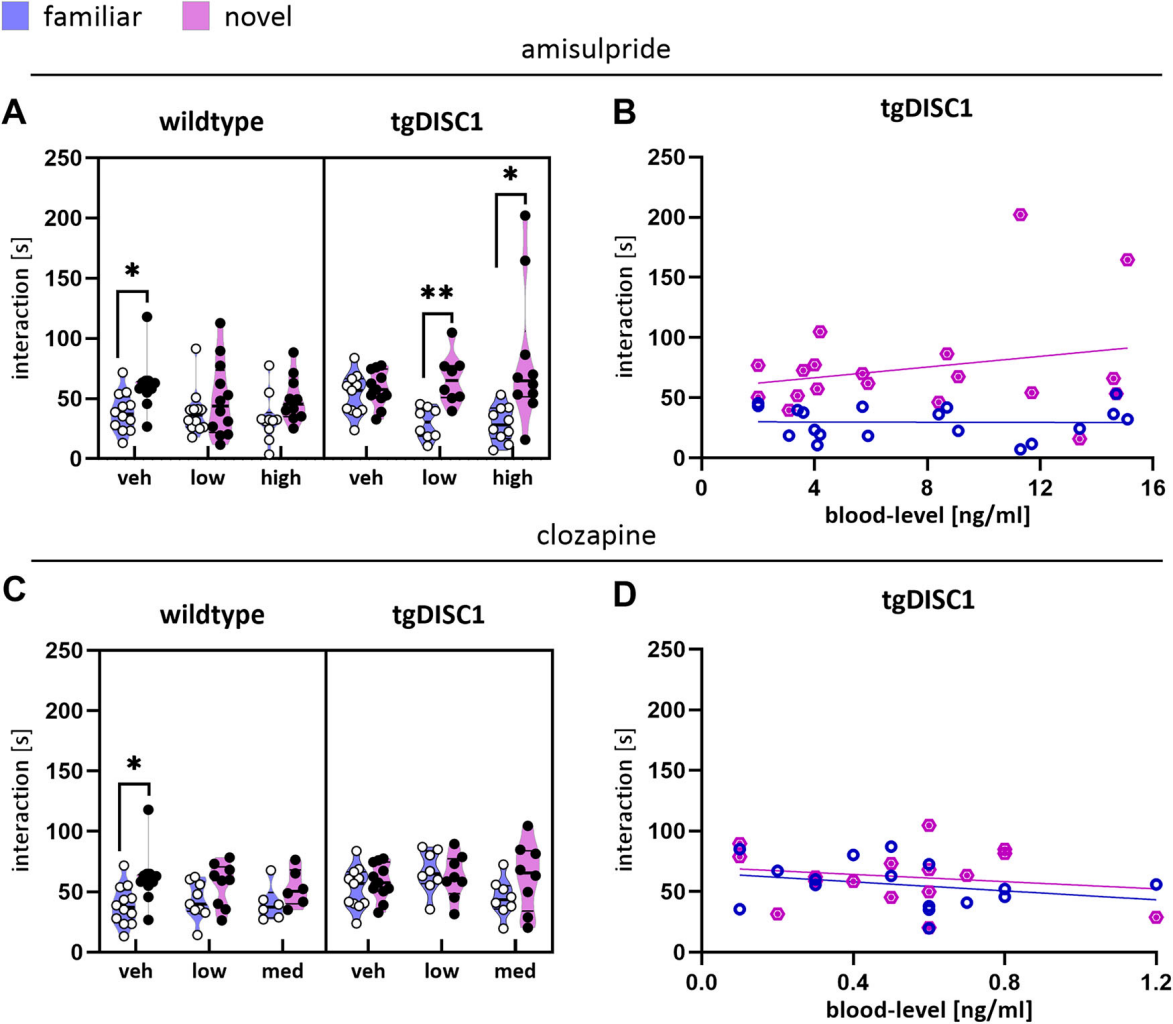
Effects of amisulpride or clozapine treatment on social novelty preference in the 3-Chamber task. **A** Social novelty preference in tgDISC1 and wildtype rats following amisulpride treatment. Social novelty preference was measured as an increased interaction duration [s] with the novel conspecific compared to a familiar one. A linear mixed-effects model revealed significant 3-way interactions (novelty * genotype* dose). Post-hoc two-sided t-test showed a significant preference for the novel conspecific in wildtype vehicles (adjusted *p* = 0.018), whereas this preference was absent int vehicle-treated tgDISC1 rats (adjusted *p* = 0.431), indicating impaired social novelty preference in tgDISC1 animals. Notably, further post-hoc tests revealed intact social novelty preference in tgDISC1 rats after treatment with either dose of amisulpride. veh = vehicle. **B** Amisulpride blood-levels did not significantly correlate with social exploration of neither the novel (diamond) nor a familiar (bold circle) conspecific in tgDISC1 rats. **C** Social novelty preference in tgDISC1 and wildtype rats following clozapine treatment. Social novelty preference was measured as an increased interaction duration [s] with the novel conspecific compared to a familiar one. A linear mixed-effects model revealed no significant effects. med = medium; veh = vehicle. **D** Clozapine blood-levels did not significantly correlate with social exploration of the novel or familiar conspecific in tgDISC1 rats. Data are presented as median ± quartiles. *p < 0.05, **p < 0.01.

For amisulpride treatment, we used a linear mixed-effects model to examine the main and interaction effects of novelty (within-subject factor), dose (between-subject factor), and genotype (between-subject factor) on social novelty preference. The analysis revealed a significant 3-way interaction at both doses (novelty * genotype * low-dose: b = -42.84 ± 21.12, t(115) = -2.03, *p* = 0.045; novelty * genotype * high-dose: b = -54.72 ± 21.27, t(115) = -2.57, *p* = 0.011). Post-hoc two-sided t-tests revealed that wildtype vehicles had a significant preference for the novel conspecific (adjusted *p* = 0.018). In contrast, vehicle tgDISC1 rats did not significantly prefer a novel conspecific over the familiar one (adjusted *p* = 0.431), confirming that tgDISC1 rats have altered social novelty preference. Notably, we found that both the low-dose and high-dose treatment of amisulpride increased social novelty preference in tgDISC1 animals (low-dose: adjusted *p* = 0.002; high-dose: adjusted *p* = 0.021, Fig. 2A). Conversely, we found no significant evidence that wildtype animals treated with either amisulpride dose preferred the novel conspecific (low-dose: adjusted *p* = 0.395; high-dose: adjusted *p* = 0.129, Fig. 2A).

Regarding the total duration of social exploration, an RLM revealed no significant main effects or dose-by-genotype interactions (all *p* > 0.263), suggesting that the effects of amisulpride were specific to novelty exploration and not general exploratory social behavior. RLMs for locomotor activity revealed dose-dependent reductions in distance traveled and average velocity, but these effects were not genotype-dependent. Specifically, amisulpride significantly reduced distance traveled at low doses in both genotypes (b = -355.74 ± 176.03, t(58) = -2.02, *p* = 0.048; interaction: b = 220.65 ± 237.80, t(58) = 0.93, *p* = 0.356). Similarly, velocity was reduced at low doses (b = -1.33 ± 0.61, t(58) = -2.18, *p* = 0.033; interaction: b = 0.97 ± 0.82, t(58) = 1.18, *p* = 0.243).

These findings highlight a genotype-dependent rescue effect of amisulpride in transgenic animals, as evidenced by increased novelty preference following treatment. This effect was absent, even inverted, in wildtype control animals, supporting a strong modulative role of amisulpride targets in shaping social adaption. Importantly, amisulpride-induced reductions in locomotor activity were dose-dependent but not genotype-dependent, suggesting that the rescue effect on social novelty exploration occurred independently of locomotor suppression. The total duration of exploration remained unaffected by amisulpride, suggesting a nuanced modulation of social behavior in transgenic rats.

In summary, we found that, compared to wildtype controls, tgDISC1 rats had significantly impaired social novelty preference which was rescued by amisulpride treatment.

The linear mixed-effects model of clozapine treated animals revealed no significant main or interaction effects on social novelty preference (all p-values > 0.061, Fig. 2C). In addition, further analysis of total exploration duration or locomotion yielded no significant results in any of the parameters measured (all p-values > 0.098). Thus, treatment with clozapine did not influence social novelty preference behavior of tgDISC1 (or wildtypes) to a significant extent.

#### Social interest

In the social interest trial of the 3-Chamber task, RLMs were used to analyze genotype- and dose-dependent effects in the time rats spent in the social chamber, interacting with the unfamiliar conspecific. No significant baseline differences in social contact between vehicle wildtype and tgDISC1 rats were revealed (wildtype vehicle vs. tgDISC1 vehicle, adjusted *p* = 0.075; descriptively, tgDISC1 rats had higher social contact durations than wildtypes).

**Fig. 3:**
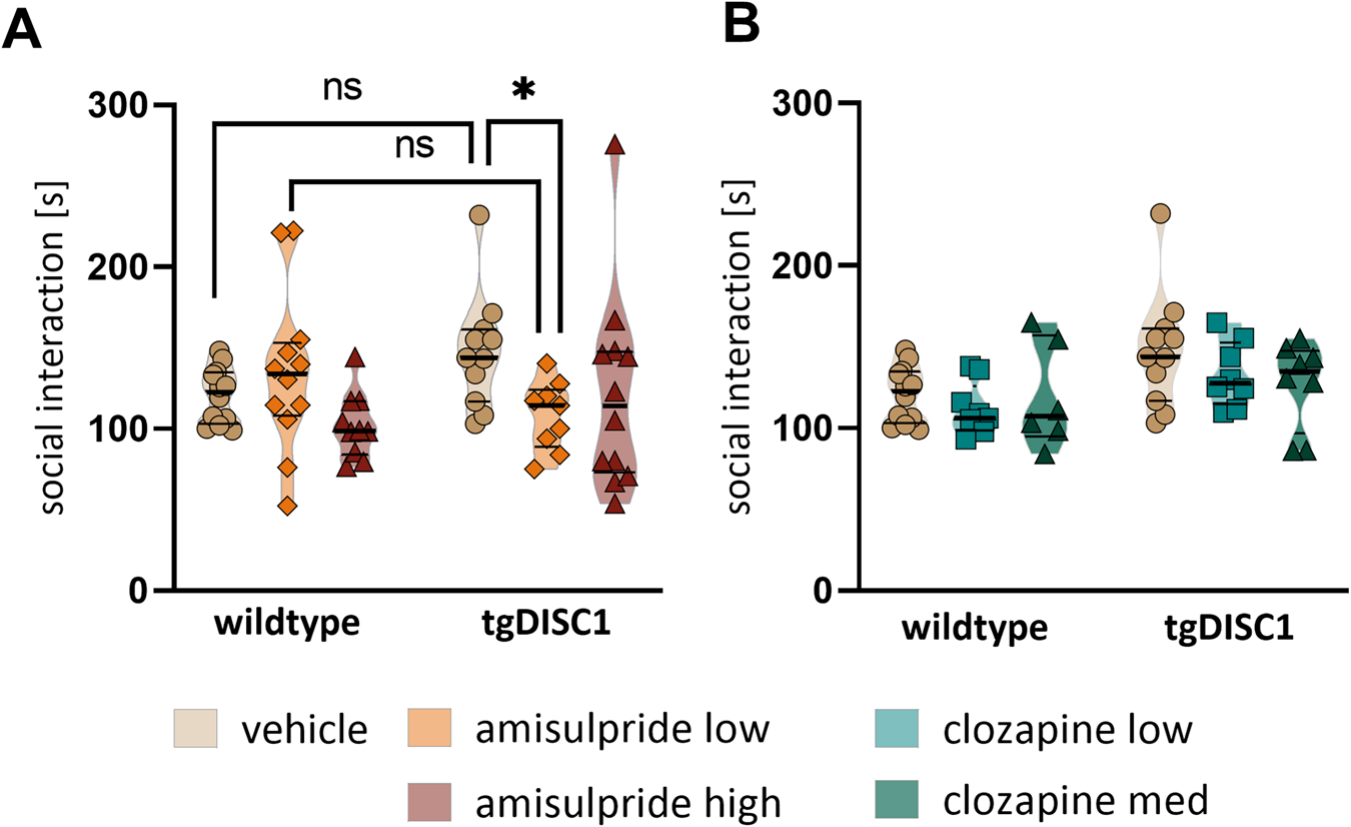
Effects of amisulpride or clozapine treatment on social interest in the 3-Chamber task. **A** Duration of social exploration during the social interest trial in the 3-Chamber task. Results of post-hoc group comparisons are visualized. ns = not significant. **B** Duration of social exploration during the social interest trial in the 3-Chamber task. No significant effects were revealed in the analysis. Data are presented as median ± quartiles. *p < 0.05, **p < 0.01.

For treatment with amisulpride, a significant dose-genotype interaction was found for the low-dose (b = 45.07 ± 18.57, t(60) = 2.43, *p* = 0.018). Post-hoc comparisons revealed that tgDISC1 rats treated with low-dose amisulpride spent significantly less time in the social chamber than the vehicle tgDISC1 rats (adjusted *p* = 0.033, Fig. 3A), while this effect was absent in wildtypes. However, a comparison between genotypes treated with low-dose of amisulpride did not show significant differences (wildtype low-dose vs. tgDISC1 low-dose, adjusted *p* = 0.096, Fig. 3A), suggesting no differences in sociability between both genotypes. For high-dose treatment, the RLM did not show a significant dose-genotype interaction effect on time spent in the social chamber (b = 11.08 ± 18.29, t(60) = 0.61, *p* = 0.544).

For clozapine-treated animals, we found no significant dose-genotype interaction effects in the social interest task. Neither low-dose nor medium-dose clozapine significantly affected social interaction in the social chamber in any group (all *p* > 0.713, Fig. 3B). However, medium-dose clozapine was associated with a significant genotype-dependent reduction in wildtypes for distance traveled (b = -562.84 ± 239.23, t(48) = -2.35, *p* = 0.023) and velocity (b = -2.03 ± 0.89, t(48) = -2.28, *p* = 0.027).

#### Sucrose preference

The Sucrose Preference task revealed no baselines differences between the vehicle groups, indicating similar hedonc drive between genotypes. Further, amisulpride had no significant effect on 10% sucrose consumption in either genotype or at low/high doses (all *p* > 0.109, Fig. S8). Similarly, clozapine did not alter sucrose intake across genotypes or doses (all *p* > 0.126, Fig. S8).

#### T-maze

In the T-maze task, amisulpride had no effect at either dose in either genotype on cognitive performance measures - score, triplets, direct/indirect revisits, and first error (all p-values > 0.085, Fig. S6B – F). Likewise, general activity readouts (arm entries, distance traveled, velocity, Fig. S6A, S6G- H) were unaffected by treatment or genotype (all p-values > 0.161, Fig. S6). For clozapine, results were similar, with no dose-dependent or genotype-related effects on any variable (triplets, direct/indirect revisits, score, first error, arm entries, distance and velocity; all p-values > 0.051, Fig. S7). Together, these findings indicate that neither amisulpride nor clozapine notably altered cognition and exploratory behavior, spatial learning, or short-term working memory performance.

#### Open field

In the Open Field task, amisulpride treatment did not influence behavioral metrics in any of the tested conditions. The RLMs revealed no significant main effects of drug dose or genotype, or their interactions, on locomotor activity, overall distance traveled, individual velocity, or number of visits to the center-zone, as a proxy for exploratory drive (all p-values > 0.531, Fig. S5). Likewise, the treatment of clozapine had no significant effects on neither genotype in any of these parameters (all p-values > 0.225, Fig. S5).

### Blood-levels

As a manipulation check, analysis of the blood-levels of the drugs revealed significant differences in drug concentrations of amisulpride between the low- and high-dose conditions, both in wildtype (t[20] = 6.62, *p* < 0.0001) and tgDISC1 rats (U = 9, *p* = 0.001, Fig. S9). The same was found in clozapine-treated rats (wildtype: t[13] = 2.55, *p* = 0.023; tgDISC1: U = 0, *p* < 0.001, Fig. S2). Further, analysis of bodyweights revealed that none of the drugs or their concentrations affected weight gain (all p-values > 0.068, Fig. S10).

#### Lack of correlations of drug plasma levels with behavioral improvement

We exploratively examined whether the behavioral task read-outs correlated with individual blood-levels of amisulpride or clozapine. Amisulpride concentrations were negatively associated with locomotor activity across genotypes in the T-maze task, as reflected in reduced distance traveled (wildtypes: r = -0.49, *p* = 0.018; tgDISC1: r = -0.46, *p* = 0.032) and velocity (wildtypes: r = -0.48, *p* = 0.022; tgDISC1: r = -0.46, *p* = 0.034). In tgDISC1 rats, higher amisulpride levels also correlated with fewer arm entries (r = -0.51, *p* = 0.017) and triplets (ρ = -0.48, *p* = 0.024). No other behavioral correlations were found for amisulpride. For clozapine, higher blood-levels were linked to reduced social interaction in wildtypes during the 3-Chamber social interest trial (r = -0.52, *p* = 0.044).

## Discussion

The present results demonstrate that social behavioral alterations in tgDISC1 rats are pharmacological modifiable by continuous treatment with the D2R/D3R antagonist amisulpride. Specifically, amisulpride restored social novelty preference in male tgDISC1 rats while leaving overall social interest, locomotor activity, and cognitive performance unaffected. This pattern indicates that the observed behavioral effects reflect a selective modulation of social adaptability – a feature often impaired in schizophrenia. in this respect, our results provide evidence that behavioral alterations in tgDISC1 rats are sensitive to antipsychotic treatment in a manner relevant to clinically used compounds, thereby directly addressing the model’s predictive validity.

The rescue of social novelty preference by amisulpride is noteworthy as it overrides seemingly hard-wired, developmental circuitry in the tgDISC1 rat^13,18^. It can be interpreted in the context of established links between DISC1 pathology and dopaminergic signaling, particularly involving D2R. DISC1 has been shown to (directly) interact with D2R and to influence their structural and signaling properties^23–26^. Additionally, they even form protein-complexes that are linked to behavioral disturbances, proving a functional relation between DISC1 and D2R expression^23–25^. In tgDISC1 rats, the overexpression of DISC1 is associated with an increase in high-affinity D2R state, which causes dysregulated dopamine homeostasis^12^. Within this framework, the ability of amisulpride – a D2R/D3R antagonist – to normalize social novelty preference may reflect modulation of dopaminergic signaling in a circuit rendered vulnerable by DISC1 protein dysfunction.

Indeed, social novelty preference has been shown to critically depend on dopaminergic signaling via dopamine D2R, placing it in a suitable context to infer pharmacological modulation of DISC1-D2R interaction^20,27,28^. In line with this, the preferential limbic affinity of amisulpride may favor its action in dopamine-rich brain regions, which we recently proved to be microstructurally altered in tgDISC1 rats^18,29,30^.

Clozapine, by contrast, may engage different pharmacological pathways that are not primarly centered on D2R/D3R modulation. Compared to amisulpride, clozapine exhibits markedly lower affinity for those receptors, while showing high affinity for D4R and a broad range of non-dopaminergic targets^31,32^. This distinct receptor-binding profile may thus act on signaling cascades that differ substantially from those engaged by more selective antagonists. For example, previous work demonstrated that clozapine shows only transient occupation and significantly lower efficacy in modulating D2R trafficking and intracellular processing compared to several other, first and second generation, antipsychotics, suggesting a reduced impact on D2R-faciliated cellular mechanisms^33,34^. This approach may be investigated by utilizing drugs showing higher affinity for D3R than D2R.

Notably, both clozapine and amisulpride display affinities for distinct serotonergic receptors, providing an additional layer of complexity when interpreting divergent pharmacological effects^21,22^. At the same time, clozapine is known to act on neurotransmitter systems not engaged by most other antipsychotics, including adrenergic, noradrenergic, muscarinic and histaminergic receptors^21^. Amisulpride, by contrast is a specific antagonist on HT-7 receptors and by that mechanism has been attributed antidepressant activity^22^. These properties raise the possibility that clozapine primary effects may preferentially manifest in physiological or behavioral domains not sufficiently captured by the paradigms applied in the presented study. Accordingly, differences in receptor profiles and downstream signaling between clozapine and amisulpride are best viewed as a contextual consideration guiding interpretation, rather than as evidence for a defined mechanistic explanation.

The observed behavior in male tgDISC1 rats adds to a distinctive phenotype, as the model shows no generalized cognitive alterations and even retains the willingness to engage with social stimuli comparable to wildtypes. By this we replicate findings that tgDISC1 rats have impairments in nuanced aspects of social behavior while broader social functioning, such as communication or interest remain intact - a critical distinction in face validity from models of severe negative symptoms^18,35,36^. Instead, they rather show a context-specific bias toward engaging with familiar individuals, thus possibly mirroring the challenges faced by individuals with schizophrenia and other psychiatric disorders, where a reluctance to engage in novel social interactions may co-exist with preserved social interest^37,38^: if patients place less value on interacting with unfamiliar individuals, this may lead to rigid or maladaptive responses to new group dynamics, which have previously been described as reduced social effort or impaired social skills in individuals with schizophrenia^39^. Importantly, various studies reported markedly improvement of social functioning and social adaption in schizophrenia patients treated with amisulpride^40–44^, strengthening the interpretation of predictive validity of the tgDISC1 model.

Our findings are consistent with the observation in human patients that behavioral improvements and motor side effects do not exhibit a straightforward correlation with plasma concentrations of antipsychotics^45^. While exploratory analyses revealed a correlation between blood-levels of amisulpride and reduced locomotion during the T-maze task, this effect was neither genotype-dependent nor sufficiently robust to yield significant differences compared to vehicle-treated groups. Overall, amisulpride demonstrated good tolerability with no persistent side effects, aligning with its established profile of a lower incidence of extrapyramidal symptoms compared to other antipsychotics^46^.

While the rescue-effect of amisulpride provides important evidence, the study holds several limitations. First, the absence of treatment effects of clozapine on the social tgDISC1 phenotype need to be interpreted with caution due to the relatively small sample size and measured blood-level concentrations, which were rather low (about 100-fold lower), compared to the concentrations considered therapeutically relevant in psychotic patients. While this discrepancy seems high, an early pharmacokinetic study of clozapine in male rats proved short half-life times of clozapine and its active metabolites, requiring continuous dosing which we addressed in this study by using Alzet pumps^47^. Contrasting with typical patterns seen in humans, continuous treatment does not lead to drug accumulation in the brain, showing pronounced species differences between humans and rats in terms of pharmacokinetics^47,48^. Likewise, a receptor occupancy study in male rats showed that continuous clozapine administration is significantly less effective than single injections, requiring up to fivefold higher doses for comparable occupancies^49^. Here, the highest clozapine dosage used was constrained by the need to maintain continuous solubility over weeks, which remained below 50 mM even in 50% DMSO, consistent with previous findings^50^. Considering those differences in drug pharmacodynamics and metabolism between species may explain the detection of low blood-levels despite accumulation in the brain.

Of note, previous literature on the usage of low-dose clozapine in male rat indicates that dosages even smaller than those used here, result in pronounced behavioral effects ^50^. In line with that, we were able to discover significant correlations of measured clozapine blood-levels and sucrose consumption in both genotypes (Fig. S1), arguing that behavioral effects were observable despite the relatively low blood-level concentrations achieved - especially since blood samples were taken temporally separated from behavioral tasks. Nevertheless, we cannot completely rule out that higher concentrations or differently administered clozapine would have led to different effects on the social phenotype of tgDISC1 rats. Given the limited and heterogeneous literature on continuous clozapine medication in animal models, further methodological standardization will be necessary to enable more direct comparison across studies and to clinically relevant treatment conditions. An additional limitation of the present study is the exclusive use of male animals.

Although social impairments have also been reported in female tgDISC1 rats, sex-dependent differences in pharmacological responsiveness cannot be excluded^20^. Considering differences in sexes regarding reported symptomatology and treatment responses^51^, future studies need to systematically address whether predicitive validity observed here also extend to female animals.

In summary, we provide converging evidence for the importance of DISC1 protein-related signaling in psychiatric characteristics – from structural to behavioral to therapeutic – making the tgDISC1 rat a valuable model in translational research. The ability of amisulpride - but apparently not clozapine - to restore social novelty preference in tgDISC1 rats highlights the centrality of dopaminergic dysregulation in their behavioral phenotype, offering a valuable target for precise therapeutic interventions in patients with DISC1-related alterations. These results also close the full circle of our precision psychiatry approach: identification of biological subsets through DISC1 aggregates^7,9^, generation of a corresponding animal model^12^, with face valid phenotypes akin to schizophrenia^12^ such as a social deficit^18–20^ and their successful pharmacotherapy (see Fig. 1). Future research should address the question, whether those results are potentially translatable back to the original patient subset.

## Materials and methods

### Animals & housing

65 male tgDISC1 rats on Sprague Dawley background and 66 of their male wildtype littermates (hereafter “wildtype”) were tested. Animals were bred at the ZETT facility (*Zentrale Einrichtung für Tierforschung und wissenschaftliche Tierschutzaufgaben*) at the Heinrich Heine University in Düsseldorf, Germany. Additionally, 24 male aged-matched wildtype Sprague Dawley rats (Janvier Labs, Le Genest-Saint-Isle, France) were used as social demonstrators for the 3-Chamber task. Rats were maintained in Type 4 cages on wood chipped bedding (LASvendi, Soest, Germany). For enrichment, a wooden block and a red PVC tube were added. Food (Ssniff, Soest, Germany) and water were provided *ad libitum*. The animals were kept at approximately 22 ± 2⁰C and 55 ± 5% humidity in a reverse 12-hour light-dark rhythm. All animal procedures were approved by the local authority LANUV (*Landesamt für Natur-, Umwelt- und Verbraucherschutz,* North Rhine-Westphalia, Germany). The final sample for analysis consisted of 97 rats after applying exclusion criteria (see Supplementary information for detailed description).

### Implantation of osmotic pumps

**Fig. 4:**
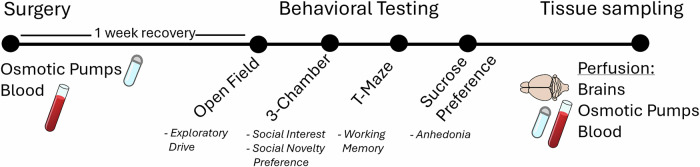
Experimental design. Shown is the experimental timeline comprising surgery and a recovery phase of 1 week, followed by sequential behavioral testing. Tissue collection was performed after completion of all behavioral assays.

After arrival at the lab, the rats were given 1 week to acclimate before the implantation surgery (Fig. 4). Animals were pseudorandomly assigned to either the vehicle group or one of the experimental groups (see below). Alzet® osmotic pumps (model: 2ML4, Durcect, Cupertino, USA) were used to ensure a steady delivery of fluid. Pumps were weighted before filling, after filling and after removal to check for residues and calculate the net injected drug. Detailed descriptions of surgical procedures, recovery, perfusion and post-mortem blood-level analysis can be found in Supplementary information.

### Experimental groups

#### Vehicle

The vehicle group received osmotic pumps filled with 2 ml saline and 50% Dimethyl sulfoxide (DMSO; Sigma-Aldrich,St. Louis, USA) as a control solution.

#### Amisulpride

Animals were pseudorandomly assigned to either a low-dose or a high-dose group. Amisulpride (CBS-FA17868, Biozol, Hamburg, Germany) solutions were prepared in 50% DMSO and saline, with the high-dose concentration adjusted to remain just below the solubility limit of 5.54 µg/µl, ensuring a delivery of 0.8 mg/kg/day based on individual bodyweights. For the low-dose, the concentration was set to 1.4 µg/µl, which corresponds to a delivery of 0.2 mg/kg/day.

#### Clozapine

Animals were pseudorandomly assigned to either a low-dose, medium-dose or high-dose group. Osmotic pumps were filled with clozapine hydrochloride (CBS-FC20526-25G, Biozol, Hamburg, Germany) diluted in Saline and 50% DMSO, with the high-dose concentration adjusted to remain just below the solubility limit (39.98 µg/µl), ensuring a delivery of 6 mg/kg/day based on individual bodyweights. For the low-dose, the concentration was adjusted to 8 µg/µl, which corresponds to a delivery of 1.2 mg/kg/day based on individual bodyweights. Note that we encountered problems in dissolving clozapine in the high-dose condition (see exclusion criteria), thus, we decided to include a medium-dose group (med-dose clozapine) with a concentration of 30 µg/µl and a delivery of 4 mg/kg/day.

#### Behavioral testing

Before the start of the experiment, the animals were brought into the experimental room for habituation for 30 minutes. Between trials, the experimental set-ups were cleaned with 70% ethanol. Experiments were conducted during the animals’ dark phase, over a course of one week. Detailed descriptions of additional control tasks and their respective exclusion criteria are provided in the Supplementary Information.

#### 3-Chamber task

The 3-Chamber offers a high translational validity for social impairments in neuropsychiatric disorders and covers multiple social domains^52–54^. First, it covers the dimension of social interest by assessing the willingness to engage in social interaction with an unfamiliar conspecific. It also measures the level of social adaptability and curiosity by quantifying the social novelty preference when an unfamiliar conspecific is introduced. The apparatus consisted of a rectangular arena (60 cm × 60 cm × 39 cm, PVC) with two compartments (20 cm × 20 cm × 39 cm) located in the left and right corners. These compartments were enclosed by bars, allowing olfactory and limited tactile interaction with conspecifics while preventing full physical contact. The task was conducted in three consecutive trials. During the habituation, the subject rat was placed in the central arena and allowed to freely explore the set-up for 5 minutes before being returned to its home cage. Following a 10-minute inter-trial interval (ITI), the social interest trial was conducted. An unfamiliar demonstrator rat was placed in one of the compartments, and the subject rat was reintroduced to the apparatus. The subject was allowed to explore and interact freely for 5 minutes before both rats were returned to their home cages. After another 10-minute ITI, the initial demonstrator was returned to its designated compartment, and a novel, unfamiliar demonstrator was placed into the remaining chamber. The subject rat was then placed back in the arena for an additional 5-minute exploration period. This is called the social novelty preference trial. All trials were recorded using a video camera (Conrad Electronic SE, Hirschau, Germany). Locomotor parameters were quantified using EthoVision XT 11.5 (Noldus, Wageningen, Netherlands). Social interaction behaviors, defined as direct contact or sniffing towards the demonstrators, were manually scored by a blinded experimenter using Solomon Coder (Solomon Coder beta 19.08.02, András Péter). Social novelty preference was measured as an increased interaction duration with the novel demonstrator compared to the familiar one. Animals were excluded from analysis of the social novelty preference trial if the subject rat did not interact with one or both of the demonstrators. Two animals were excluded from analysis.

### Statistical analysis

Statistical analyses were performed using RStudio (v2023.06.2), with significance set at *p* < 0.05. Normality of the data was assessed using the Kolmogorov-Smirnov test. Depending on distribution, group comparisons used unpaired t-tests or Mann-Whitney U test. Pearson or Spearman correlations were applied based on normality. Robust linear-models (RLM) assessed main and interaction effects of multiple independent variables and linear mixed-effect models where used for repeated measures. When post-hoc pairwise comparisons were necessary, the Benjamini-Hochberg false discovery rate (FDR) procedure was applied to adjust for multiple comparisons. Statistical figures were created using GraphPad Prism 9.5.0 (GraphPad Software, Boston, USA).

## Supplementary information

Supplementary Material

## Data Availability

All data that support the findings of this study are available upon request.
