# Supplementary material for: Pharmacological rescue of social deficits in rats featuring Disrupted-in-Schizophrenia-1 (DISC1) protein aggregation
Source: Schizophrenia (Heidelb). 2026 Feb 4;12(1):16. doi: 10.1038/s41537-026-00729-y (PMC12877177; doi:10.1038/s41537-026-00729-y)
Supplement: Supplementary file 1 — Supplementary Material [file 41537_2026_729_MOESM1_ESM.pdf]

Pharmacological rescue of social deficits in rats featuring Disrupted-in-Schizophrenia-1 (DISC1) protein aggregation

José Dören[1], Else Van Gerresheim[2], Sandra Schäble[1], Svenja Troßbach[2], Ann-Christin Langen[2,3], Heike Schneider[4], Werner Steimer[4], Tobias Kalenscher[1*], Carsten Korth[2*]

Supplementary Materials

Supplementary Materials and Methods

Genotyping & Re-Genotyping

The procedure for genotype assessment was following established qPCR protocols (Trossbach et al., 2016). After termination of animals, we performed a re-genotyping to ascertain correct group assignment.

Surgery & Recovery

Prior to surgery, rats received analgesia (5 mg/kg carprofen s.c.). For anesthesia, inhalation was induced with 5% isoflurane until rats lost mobility, and then, isoflurane levels were lowered to 2% to 3% for maintaining anesthesia. Upon reaching surgical state, rats were fixed with their teeth into a stereotactic apparatus (David Kopf Instruments, Los Angeles, USA).

The fur was shaved on pseudorandomly assigned left or right dorsal flank and a ca. 2cm long incision was made for subcutaneous placement of the osmotic pump. The wound was closed with stitches and treated with antiseptic spray. In addition, blood samples were collected. Bodyweights were taken the day of the surgery as well as the first and second day following. Rats had one week of recovery before the start of the behavioral experiments. One rat died during surgery.

Perfusion & Tissue Preparation

Approximately three weeks (21 – 24 days) after receiving the pumps, rats were terminated. At termination, rats were injected with pentobarbital and deeply anesthetized with isoflurane until reaching surgical state. Blood was drawn to check blood-level concentration of drugs. Following, half of the rats had their brains removed that got immediately snap frozen in Isopentane.

The other half of the rats was perfused transcardially with 4% paraformaldehyde in 0.1 M phosphate buffer. Brains were immediately removed (and snap frozen) and stored at -80°C. Osmotic pumps were removed and weighted to check for any residuals of leftover drugs and to calculate the net amount injected over the implanted period.

Post-mortem Blood-level Analysis

Amisulpride and clozapine were quantified by isotope dilution mass spectrometry (IDMS) using an AB Sciex QTrap 5500 (AB Sciex™ DH Tech., Framingham, MA 01701, US) with a Shimadzu prominence HPLC-System (Shimadzu, Duisburg, Germany) as front end. Method was adapted to the method previously published (Kirchherr & Kühn-Velten, 2006). In short, 10 µl of serum was mixed with 40 µl water and 150 µl precipitation solution (70 % methanol, 30 % acetonitrile (v/v)) containing internal standards. Internal standards were fluoxetine D5 (Toronto Research Chemicals Inc. North York, Kanada and amisulpride D5 (Alsachim;

Shimadzu Chemistry & Diagnostics, Illkirch Graffenstaden, France). 5 to 10 µl of supernatant (10000*g, 10 minutes) were injected into the LCMS system. Separation was carried out on an Onyx C18 column (100 x 3.0 mm; Phenomenex, Aschaffenburg, Germany) by gradient elution. Starting with 90 % mobile phase A: water purified by Milli-Q® IQ 7000 purification system (Merck Chemicals GmbH Darmstadt, Germany) buffered with 0,1 % acetic acid and 12,5 mM NH_4OAc (both Merck KGaA, Darmstadt, Germany) changing to 100 % mobile phase B: LCMS-grade methanol (Th. Geyer GmbH & Co. KG. Renningen, Germany) buffered equal to mobile phase A. After 5 minutes concentration was set back to 90 % phase A. Total run time was 6.5 minutes. Quantification was performed by peak integration in MRM mode. 3PLUS1® Multilevel Plasma Calibration Set Neuroleptics 1 and 2 (Chromsystems GmbH, München, Germany) were utilized. Mass transition 370.2/242.0 was used to quantify amisulpride and 327.2/192.1 to quantify clozapine. Quantification was controlled by MassCheck® Neuroleptics 1 Plasma Control and MassCheck® Neuroleptics 2 Plasma Control, two levels each (Chromsystems GmbH, München, Germany). With the selected dilution a lower limit of quantification (LOQ) of 0.5 ng/ml for amisulpride and 0.1 ng/ml for clozapine was achieved.

Exclusion Criteria

Despite reports of sufficient clozapine solubility in DMSO (Huang et al., 2021) at the highest concentrations used (6 mg/kg/day in 2.2 ml saline and 50% DMSO), clozapine formed visible, insoluble precipitates over the three-week treatment. This resulted in incomplete emptying of the pump (weighed at termination), indicating no diffusion into the rat bodies.

Individuals were excluded from further analysis if their blood-levels of amisulpride or clozapine were below 0.1 ng/ml (i.e. detection limit). Initial checks revealed that all nine animals tested until then that received the highest clozapine dose (6 mg/kg/day) met the exclusion criteria due to precipitates or undetectable blood levels, leading us to discontinue the high-dose treatment. After completing data collection, we found that seven of 24 rats in the low-dose group (1.2 mg/kg/day) and ten of 24 in the medium-dose group (4 mg/kg/day) also met the exclusion criteria and were subsequently removed from analysis. Furthermore, six rats were excluded due to incorrect genotyping (heterozygotes), and one rat was terminated after meeting termination criteria.

Behavioral Testing

<u>Open Field</u>

An Open Field test was conducted to assess potential differences in locomotion and exploration between the groups. Rats were placed in the center of a square arena (50 cm x 50 cm x 45 cm, PVC)

and could freely explore it for 10 min while being recorded by a camera (Conrad Electronic SE, Hirschau, Germany) from above.

The tracking software EthoVision XT 11.5 (Noldus, Wageningen, Netherlands) was used for behavioral read-outs. The number of visits to the center zone were used as a measure for exploratory drive and the total distance moved as well as the average velocity of an individual were measured to assess locomotor activity and potential deficits.

T-Maze

To assess short-term working memory and (rigid) pattern exploration, we utilized a T-maze task. The T-shaped apparatus consisted of three arms (57 cm × 16 cm × 37 cm each, PVC) and a center zone (37 cm x 37 cm x 37 cm, PVC). For distinction, each arm of the T-maze had unique visual cues on the walls. The animals were placed in the center zone facing a wall. For a period of five minutes, the animals could explore the maze freely and the order of arm entries were noted. The trials were recorded (Conrad Electronic SE, Hirschau, Germany). Parameters measured were numbers (#) of: total arm entries, triplets, direct re-visits, indirect re-visits, number of the first error entry, as well a performance Score. The Score was calculated as follow:

$$Score = \frac{\#\ triplets}{(\#\ total\ entries\text{-}2)}$$

In addition, EthoVision XT 11.5 (Noldus, Wageningen, Netherlands) was used to measure total distance travelled and average velocity as locomotion readouts.

Sucrose Preference

The Sucrose Preference Task assesses anhedonic phenotypes by measuring the preference for a sucrose solution over water (Scheggi et al., 2018). Animals were single housed the night prior to the test and had free access to water and food by distributing food pellets into the cage. The following morning, a 10% sucrose solution (Roth, Karlsruhe, Germany) was prepared. The bottles containing the sucrose and an additional bottle of water were placed in a random order on the cage lids. The weight of the bottles was measured before presenting them to the animals and after 6 hours. After the 6-hour period, rats were put back into their home cage. The weight measurements were analyzed. Individuals were excluded from analysis if any of the presented bottles happened to spill. Seven animals were excluded from analysis.

Figure S1 Methods. This task-design is based on a previous publication about the social phenotype in the tgDISC1 rats (Seidisarouei et al., 2022). Here, we made an attempt to adapt a one-shot version of the paradigm, challenging different motivational states by presenting two different reward types at the same time. Thus, we were able to compare the time allocation of rats in each reward zone. In brief, during a 10-minute trial, rats had the chance to freely explore a rectangular apparatus (154 cm x 16 cm x 37 cm, PVC). In the far ends, either a bottle containing 10% sucrose solution or a restrainer, holding an unfamiliar conspecific of the same age and sex, were placed. The restrainer was made out of bars, allowing olfactory and limited tactile interaction with the conspecific while preventing full physical contact. The duration of social interaction and time spent drinking the sucrose solution were manually scored. In addition, bottles containing the sucrose solution were weighed before and after the experiments to determine the amount of consumption. Note that rats of each genotype treated with the vehicle compound preferred social contact over sucrose consumption and thus did not differ in their time allocation between the two rewards.

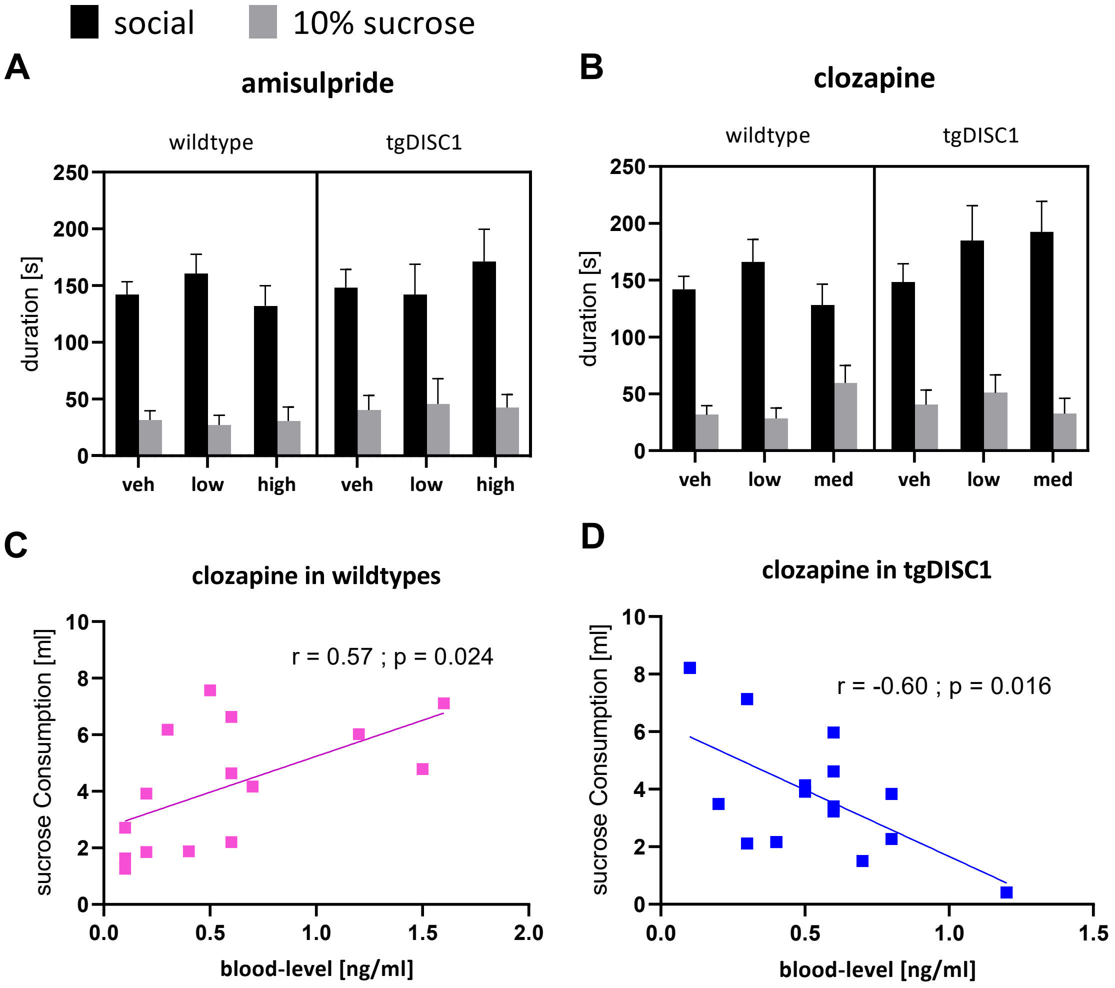

Figure S1: Comparison of different reward types in wildtype and tgDISC1 rats. During the task, rats had the chance to freely explore an apparatus which far ends offered either a bottle containing 10% sucrose solution or the interaction with an unfamiliar conspecific of the same age and sex. Data is show as mean ± standard error of mean (SEM). (A) Duration spent in each reward zones. Amisulpride treatment did not significantly affect behavior of either genotype. (B) Duration spent in each reward zones. Clozapine treatment did not significantly affect behavior of either genotype. (C) Correlation of clozapine blood-levels and sucrose consumption in wildtypes. Higher blood-levels of clozapine were significantly correlated with higher sucrose consumption. (D) Correlation of clozapine blood-levels and sucrose consumption in tgDISC1. Higher blood-levels of clozapine were significantly negatively correlated with sucrose consumption.

Supplementary Results

3- Chamber

Social Interest

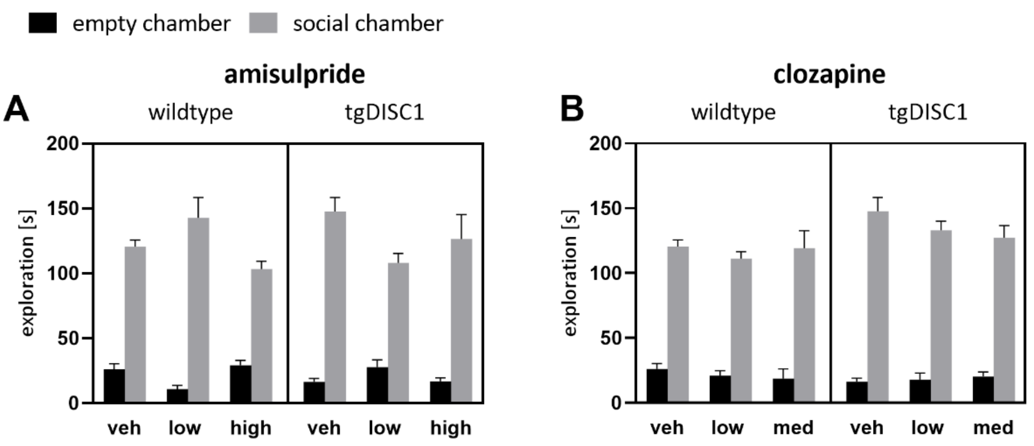

Figure S2. Comparison of exploration time across chambers in the three-chamber social interaction apparatus in wild-type and tgDISC1 rats. Total duration spent in each chamber is shown. (A) Continuous treatment with amisulpride at low or high dose did not significantly alter chamber exploration in either genotype. (B) Continuous treatment with clozapine at low or medium dose did not significantly alter chamber exploration in either genotype. Data are presented as mean ± SEM.

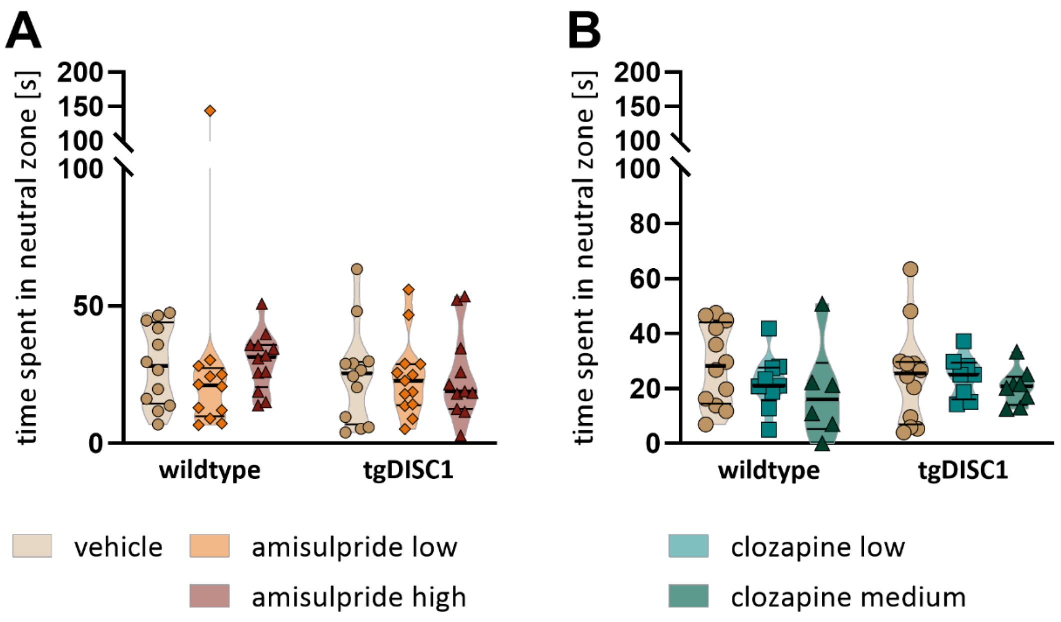

Figure S3. Comparison of time spent in the neutral zone during the social interest trial in wild-type and tgDISC1 rats. (A) Time spent in the neutral zone was not significantly altered by low or high doses of amisulpride in either genotype. (B) Time spent in the neutral zone was not significantly altered by low or medium doses of clozapine in either genotype. Data are presented as median ± interquartile range.

Social Novelty Preference

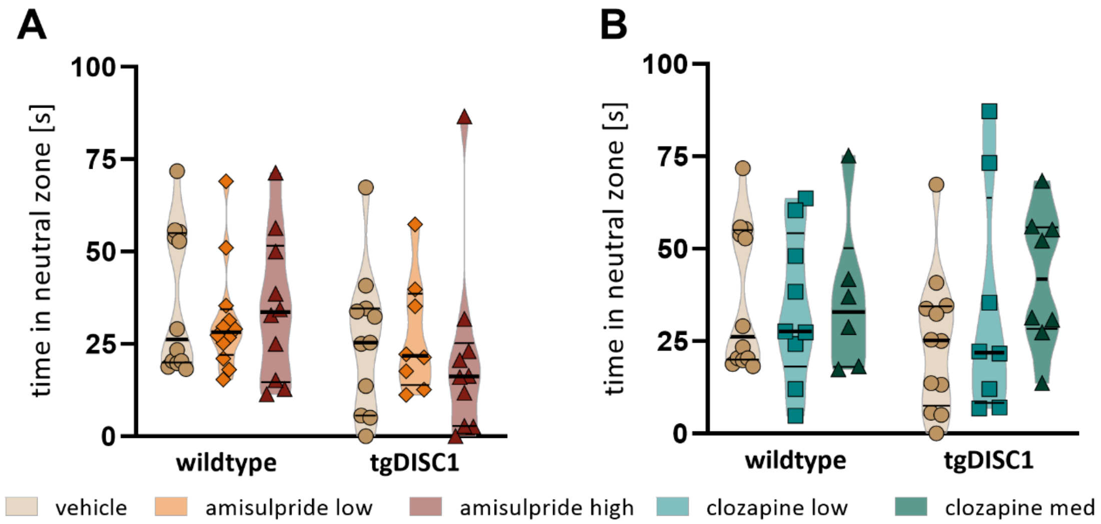

Figure S4. Comparison of time spent in the neutral zone during the social novelty preference trial in wild-type and tgDISC1 rats. (A) Time spent in the neutral zone was not significantly altered by low or high doses of amisulpride in either genotype. (B) Time spent in the neutral zone was not significantly altered by low or medium doses of clozapine in either genotype. Data are presented as median ± interquartile range.

Open field

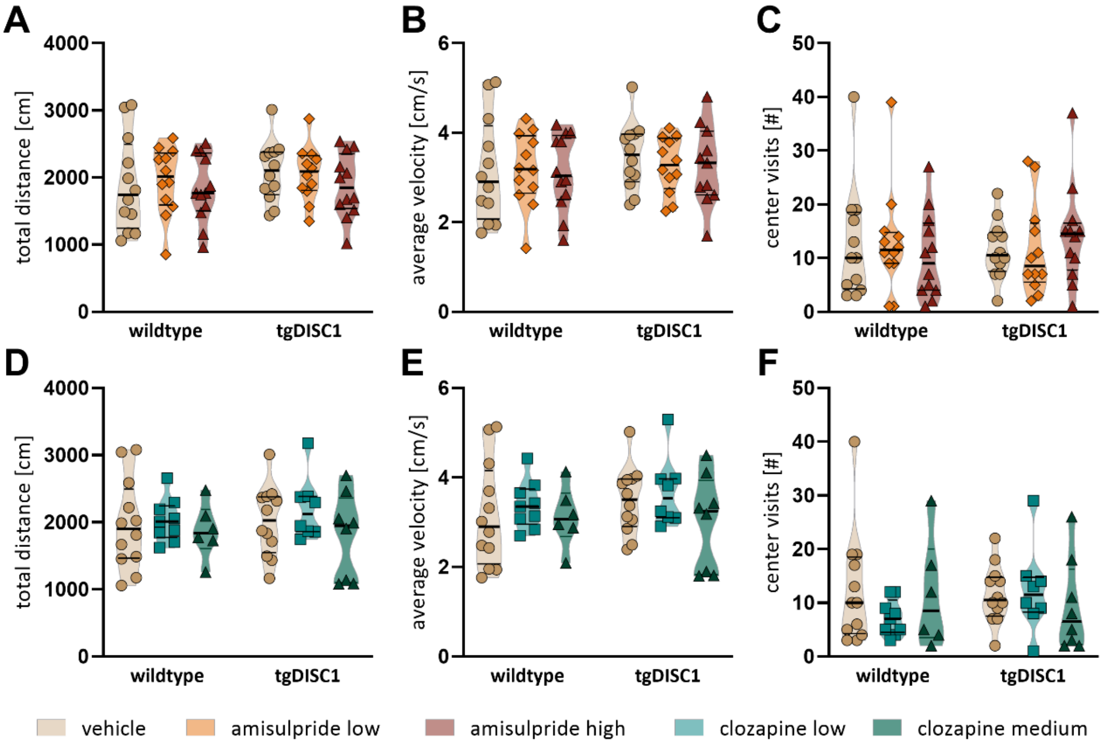

Figure S5. Comparison of behavioral readouts in the open field test in wild-type and tgDISC1 rats. (A–B) Locomotor performance was not significantly altered by low or high doses of amisulpride in either genotype. (C) Exploratory drive, assessed by the number of visits to the center zone of the arena, was not significantly altered by low or high doses of amisulpride in either genotype. (D–E) Locomotor performance was not significantly altered by low or medium doses of clozapine in either genotype. (F) Exploratory drive, assessed by the number of visits to the center zone of the arena, was not significantly altered by low or medium doses of clozapine in either genotype. Data are presented as median ± interquartile range.

T-Maze

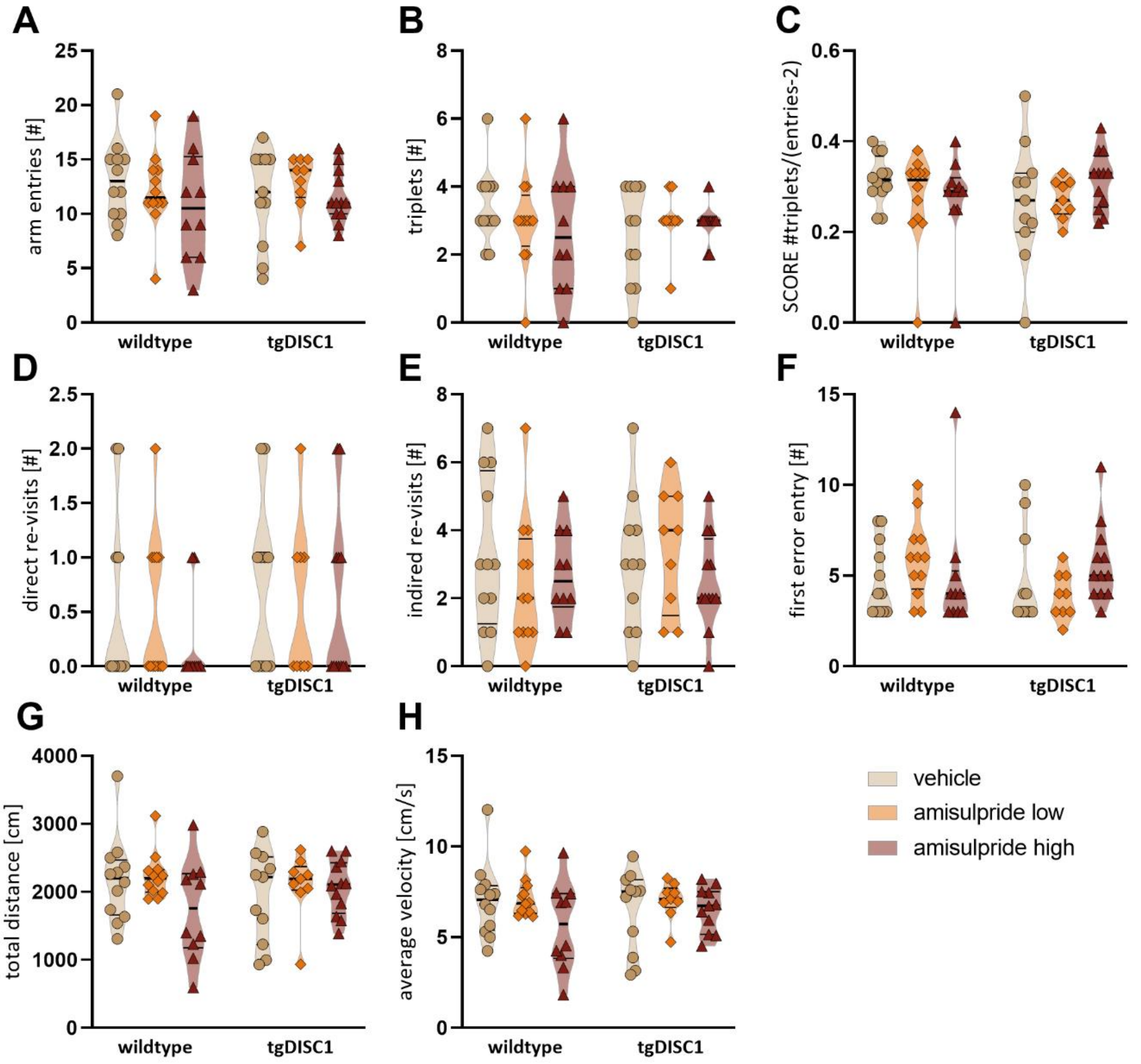

Figure S6. Comparison of behavioral readouts in the T-maze task in wild-type and tgDISC1 rats. (A–F) Cognitive performance in the T-maze task was not significantly altered by low or high doses of amisulpride in either genotype. (G–H) Locomotor performance in the T-maze was not significantly altered by low or high doses of amisulpride in either genotype. Data are presented as median ± interquartile range.

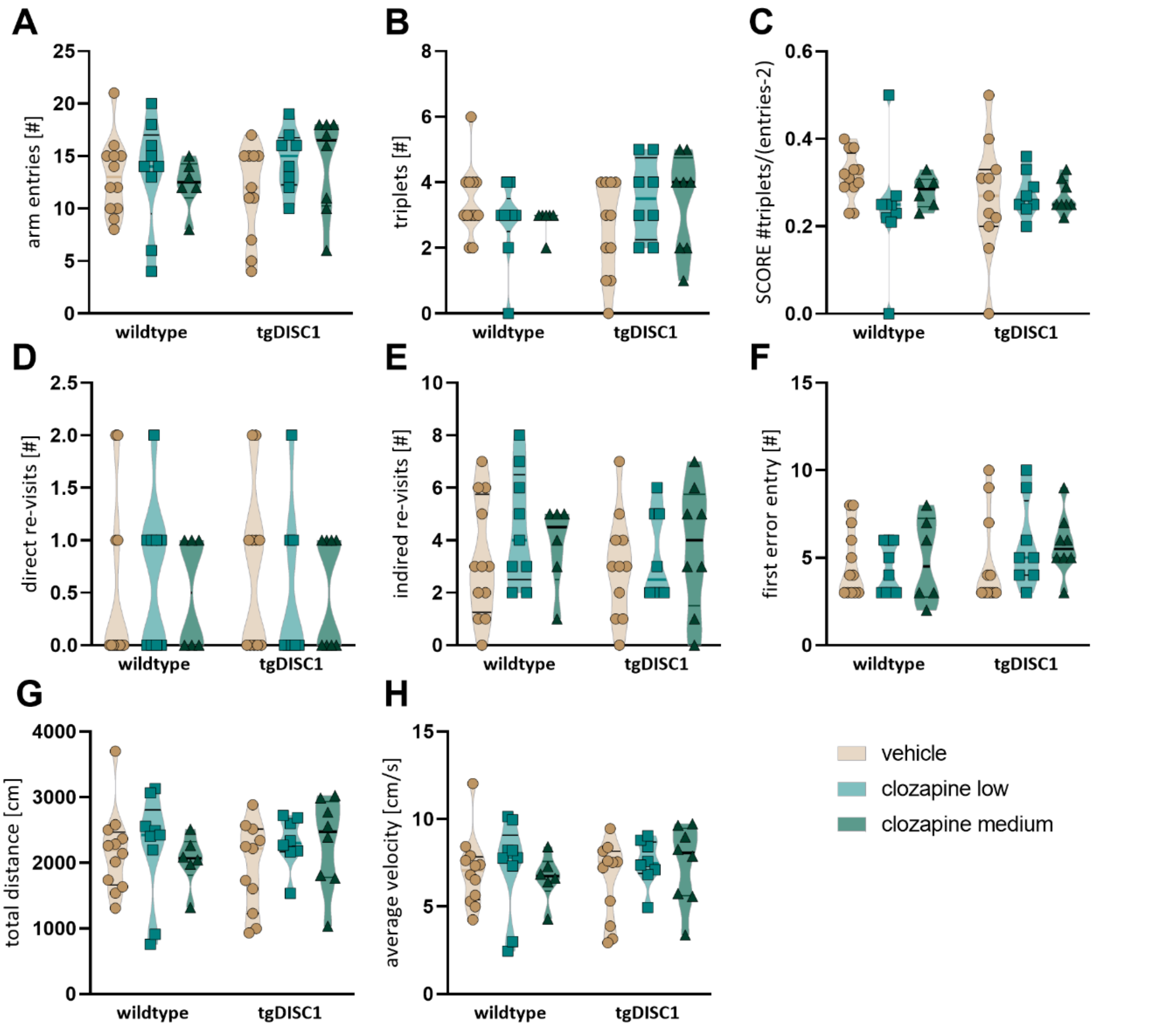

Figure S7. Comparison of behavioral readouts in the T-maze task in wild-type and tgDISC1 rats. (A–F) Cognitive performance in the T-maze task was not significantly altered by low or medium doses of clozapine in either genotype. (G–H) Locomotor performance in the T-maze was not significantly altered by low or medium doses of clozapine in either genotype. Data are presented as median ± interquartile range.

Sucrose Preference

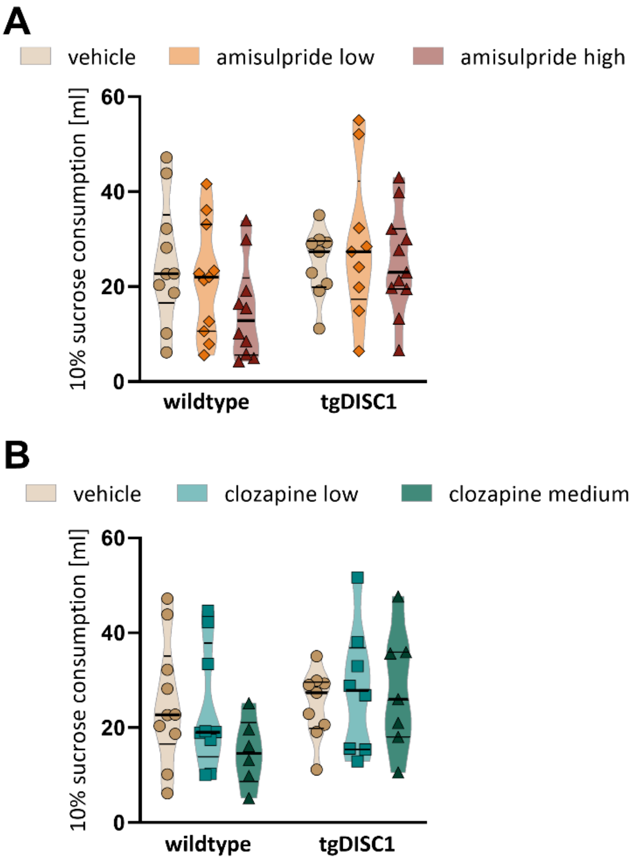

Figure S8. Comparison of consumption of a 10% sucrose solution in wild-type and tgDISC1 rats. (A) Consumption of the 10% sucrose solution was not significantly altered by low or high doses of amisulpride in either genotype. (B) Consumption of the 10% sucrose solution was not significantly altered by low or medium doses of clozapine in either genotype. Data are presented as median ± interquartile range.

Blood-levels and Bodyweights

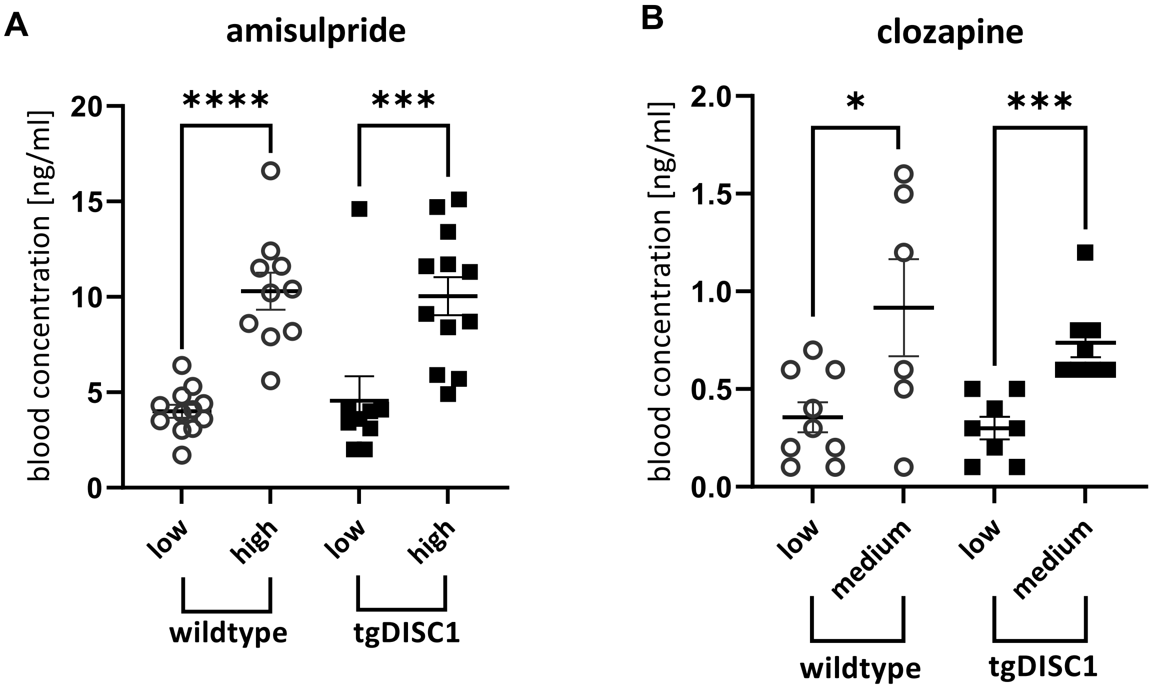

Figure S9: Blood-levels of amisulpride and clozapine, in wildtype and tgDISC1 rats. (A) Blood concentration levels (mean ± SEM) of amisulpride in wildtype and tgDISC1 rats following low- and high-dose treatment. The blood concentrations differed significantly between the low- and the high-dose conditions in both genotypes (wildtype: $p < 0.0001$; tgDISC1: $p = 0.001$). (B) Blood concentration levels (mean ± SEM) of clozapine in wildtype and tgDISC1 rats following low- and medium-dose treatment. The blood concentrations differed significantly between the low- and the medium-dose conditions in both genotypes (wildtype: $p = 0.023$; tgDISC1: $p < 0.001$). *$p < 0.05$, **$p < 0.01$, ***$p < 0.001$

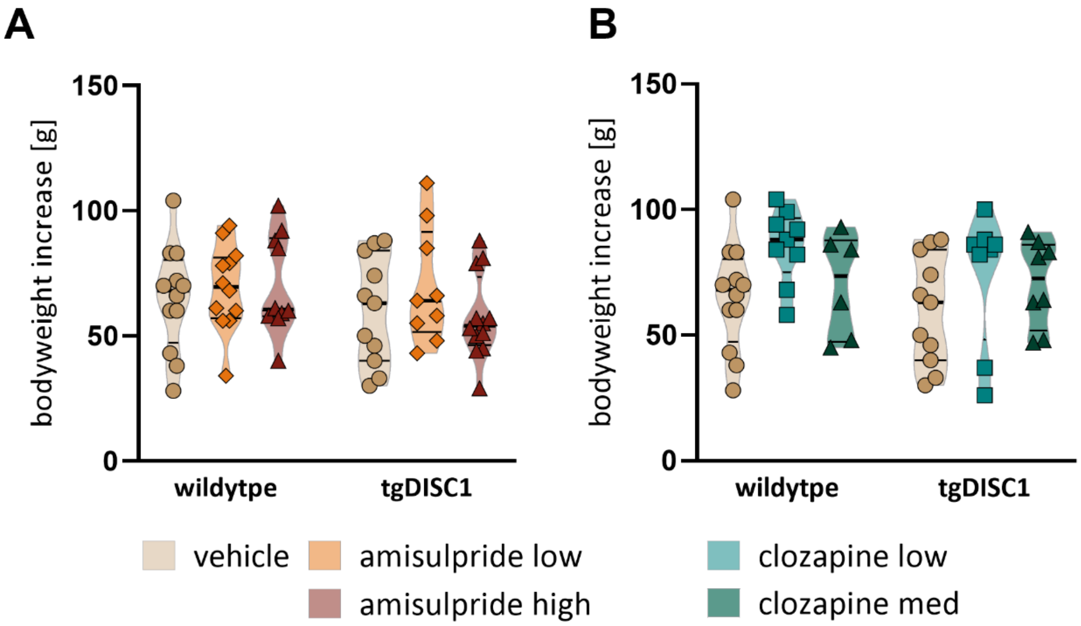

Figure S10. Comparison of body weight gain in wild-type and tgDISC1 rats. (A) Body weight gain was not significantly altered following low or high doses of amisulpride in either genotype. (B) Body weight gain was not significantly altered following low or medium doses of clozapine in either genotype. med = medium. Data are presented as median ± interquartile range.

References

1. Trossbach, S. V. *et al.* Misassembly of full-length Disrupted-in-Schizophrenia 1 protein is linked to altered dopamine homeostasis and behavioral deficits. *Mol Psychiatry* **21**, 1561–1572 (2016).
2. Kirchherr, H. & Kühn-Velten, W. N. Quantitative determination of forty-eight antidepressants and antipsychotics in human serum by HPLC tandem mass spectrometry: A multi-level, single-sample approach. *Journal of Chromatography B* **843**, 100–113 (2006).
3. Huang, W. *et al.* Measurement and correlation of solubility, Hansen solubility parameters and thermodynamic behavior of Clozapine in eleven mono-solvents. *J Mol Liq* **333**, 115894 (2021).
4. Scheggi, S., De Montis, M. G. & Gambarana, C. Making Sense of Rodent Models of Anhedonia. *International Journal of Neuropsychopharmacology* **21**, 1049 (2018).
5. Seidisarouei, M. *et al.* Social anhedonia as a Disrupted-in-Schizophrenia 1-dependent phenotype. *Sci Rep* **12**, (2022).
